# The Future Science of Medicine*Reprinted from Monthly Cyclopedia of Practical Medicine, Vol. X, page 385, 1907.

**Published:** 1907-12

**Authors:** J. Madison Taylor

**Affiliations:** Philadelphia


### The Future Science of Medicine.*

BY J. MADISON TAYLOR, A. B., M. D., PHILADELPHIA.

Under the above heading the *St. Louis Medical Review* of June 8, 1907, published the following lines: "Dr. C. E. de M. Sajous announced on June 3d, at the American Medical Editors' Association, the crowning point of his patient labors on the ductless glands, in the discovery in the pituitary body of a membrane functionally resembling the Schneiderian [its olfactory area], in that it tested the condition of the body fluids and automatically regulated the correction of depraved conditions by producing antitoxins. Complete details by the author will appear shortly. It seems probable that an *absolutely scientific therapy* is now within sight." The announcement referred to was made at the dinner of the Association, which took place on the day mentioned. The complete details are to be found in the second volume of Sajous' "Internal Secretions," which has since appeared. The great interest awakened by his address, and the recent announcement that "Internal Secretions" was regarded on the continent of Europe as so marked an advance in our knowledge of the functions of the ductless glands that it is to be translated into French by one of the greatest authorities on the anatomy and histology of these organs, Professor Launois, of Paris, who has suggested the advisability of giving our readers an outline of the function referred to above, which will unquestionably revolutionize medicine in the sense specified by the *St. Louis Medical Review.*

As is now generally known, Sajous' study of the functions of the ductless glands was only an incidental feature of his purpose to give medicine a more solid foundation than that upon which it rests at the present time. Nearly twenty years ago, when, as editor of the *Annual of the Universal Medical Sciences,* it became his duty to collate yearly the progress in all branches of medicine, he was surprised to note the amount of theorizing being indulged in by investigators in every branch of medical science: physiologists, physiologic chemists, histologists, therapeutists, clinicians, etc. After reciting a few experiments on clinical observations, and giving a perfunctory and often very imperfect review of the literature of the subject, authors of unquestionable merit would not hesitate to launch forth tentative deductions on every conceivable sub-

*Reprinted from Monthly Cyclopedia of Practical Medicine, Vol. X, page 385, 1907.

ject, until our knowledge of any question became literally congested with discordant figments of imagination. Whether these new theories might not become flagrant misfits when everything became ultimately known of the questions to which they were appended was not taken into account of their authors; they had launched *"something original,"* and on the strength of the frogs and guinea pigs used in the experiment, or the few clinical observations quoted, and that something was assured by them to be immensely "scientific." It is to this fundamental defect that Sajous ascribes the present deplorable condition of medicine, which he compares to that of art during the Dark Ages. What has been justly termed by an editorial writer in the *New York Medical Record* "Osler's black, hopeless, helpless, therapeutic pessimism" has, in his opinion, no other cause. He holds that if we are forced to admit today that Skoda's well-known dictum that "we can diagnose disease, describe it, and get a grasp of it, but we dare not expect by any means to cure it" still holds good, it is because much that is valuble in the work of modern investigators is hidden under the maze of false and misleading conclusions with which they have encumbered medical lore.

How can confidence in medicine as the "healing art" be restored? Can it be achieved through the sacrifice of yet more frogs, more guinea pigs, by more laboratory guesses—the addition of a few more theories to the thousands that have driven medicine to practical bankruptcy? Sajous concluded that but one course afforded any chances of success in this direction, viz., to cast aside all theories, and with the aid of the huge aggregation of positive facts, experimental and clinical, actual results, etc., recorded by reliable investigators in all branches of medical science, *seek the solution of all admittedly undiscovered functions* even as a mathematician deals with a series of problems which he may wish to solve. By this plan, he avoided entirely the pit into which investigators had hitherto fallen, i. e., that of being inspired by any preconceived theory, while giving all experimental and clinical facts their legitimate place in the process—e. g., the place filled by the bricks, stones, wood, mortar, metals, etc., used in the erection of a building. This involved the use of logic, i. e., of analytic and synthetic reasoning, which, according to Sajous, are utilized too sparingly by modern investigators. The great French biologist, Milne-Edwards, wrote many years go: "The history of science teaches us to do justice to the modest investigators whose patient labors have furnished us the material thanks to which *generalizing minds* have been able to construct the scientific edifice. But, above all, it teaches us to appreciate those men who, avoiding vain speculations, and reasoning only from well-established facts, have been able to encompass the aggregate of these phenomena within their field of vision and point to the general and constant relations which unite them one to the other." That synthetic philosophy is a *sin qua non* of this, the highest and most difficult mission which any scientific man can undertake, is abundantly obvious.

It is to his *analytic* work that Sajous owed the discovery that the underlying cause of the existing confusion in medicine was due to the prevailing lack of knowledge concerning the functions of the ductless glands; it was his *synthetic* work which led him to the discovery of the true rôle of these organs in the body. As soon as these functions had been established by him, *hundreds* of problems, ninety-six of which he enumerates in the introduction to his second volume (and any one of which would qualify him to earn the gratitude of posterity), found a ready solution, the experimental results of a multitude of investigators thus falling into line, as it were, of their own accord. Pulmonary and tissue respiration, absorption and nutrition, the circulation of the nervous system (Harvey having discovered that of the larger vessels and Malpighi that of the capillaries), the nature of organic function and the manner in which it is awakened by vasodilator nerves, the composition of ferments, the physiologic and morbid production of sleep, etc., are but a few of the many problems which physiologists had admittedly failed to solve, as is well illustrated by Osler's remark that while we know little concerning the action of drugs, "we put them into bodies the action of which we know less."

When once all these problems were solved, and the solutions proven correct by the precision with which they all harmonized, a superb mechanism revealed itself to Sajous: that of the human organism *complete,* the functions of the ductless glands and the presence of their products in all organs having filled many deplorable gaps—those identical functions which physiologists and histologists, notwithstanding their painstaking labors, had failed to explain. The tendency of modern investigators to introduce hypotheses, tentative guesses, etc., on all topics was also explained: they had observed phenomena on all sides which, without a knowledge of the functions of the ductless glands, were unintelligible, and for which they supplied what appeared to them as plausible explanations.

A brief review of the main steps of Sajous' labors will not only serve to illustrate all these facts, but it will lead up to the crowning feature of his work, viz., the discovery of the process through which the body antagonizes disease by providing the blood with what he has termed its "auto-antitoxin":

*Adrenals.*—Sajous found that these organs supplied a secretion which passed to the lungs and took up therein the oxygen of the air. This solved the cardinal problem of human functions: that of pulmonary respiration. Physiologists had also failed to discover the identity of 94 per cent of the hemoglobin molecule. This likewise proved to be the oxygenized adrenal secretion. The nature and the source of an oxidizing substance found in the blood, oxidase, had also remained undetermined. Sajous found that this substance, the (albuminous) 94 per cent of hemoglobin, and the oxygenized adrenal secretion were one and the same; that all tissues contained it; and that it was this substance which supplied the tissues with oxygen. He discovered another important fact in this connection, viz., that it was the adrenal active principle, thus distributed to all cells, which sustained their life—the principle which Herbert Spencer deemed necessary to account for the vital process (his "dynamic element of life"), but the presence, source, and identity of which were to him unknown. Dr. Sajous had thus solved simultaneously the problems of tissue respiration and cellular life. The importance of these discoveries from the standpoint of practice can not be overestimated, for, as we will now see, we are able *with our remedies* to *govern* the adrenals, and, therefore, *oxygenation* of all cells and the *life process* itself where its activity is subnormal.

*Thyroid Gland.*—As is now well known to all physicians, thyroid extract given to a cretin or an idiotic child in whom the functions of the thyroid glands are deficient, brings about a wonderful change. The body soon begins to grow, all the functions are remarkably stimulated, and the brain, practically inactive before, assumes its physiologic rôle as the organ of thought. What amounts to a mere "human plant" is finally transformed into a normal child, and remains such, but only so long as thyroid extract is administered to it. Now, thyroid extract has long been known to enhance actively the body's oxygenation. But how does it bring about this result? How does it produce the wonderful transformation in the cretin? Sajous also solved this problem. He found that *the purpose of the thyroid secretion was to excite a center in the brain* (to which reference will be made presently) connected with the adrenals by nerves, and that it was, therefore, by stimulating indirectly the adrenals that the thyroid extract produced its wonderful effects.

It is here that the great practical importance of Sajous' discoveries is demonstrated. Not only did he find that thyroid extract excited the adrenal center, but that several of our remedies, iodine, the iodides, mercury, coca, and others, did likewise. The unexplained physiologic action of these remedies in combating some of the most destructive diseases of mankind thus became clear; they increased the oxygenizing power of the blood and the activity of the vital process, and thereby the power of the body to fight disease and destroy pathogenic bacteria, the poisons they secrete, toxic wastes, etc.

The thyroid gland was also found by Sajous to be the source of a substance which has been receiving considerable attention of late —Wright's "opsonin," known to sensitize bacteria and render them vulnerable to the attacks of phagocytes—those white cells or leucocytes of the blood and lymph which act as the body's scavengers and do so much to protect it against infectious diseases, as shown by Metchnikoff. This introduces another practical point of the highest importance connected with Sajous' discoveries, viz., the functions of the leucocytes.

*The Leucocytes.*—The white cells of the blood have been given several different rôles by physiologists, but Sajous was the first to show their true function: the identity of that which causes them to act as scavengers; to appear in great numbers in the blood under certain conditions, normal and morbid; to contain numerous digestive ferments, etc. He showed that their rôle in the body was to take up or "engulf" food products of any kind, both in the intestinal canal (after the foods had been partially digested in the stomach and intestine) and in the blood and other body fluids; to convert these food products into *living* granulations (through the adrenal principle which their ferments contain), and to transport them to the tissue cells. Sajous thus contributed another great advance in our knowledge of cell-life: not only did he show the identity and source of the dynamic principle which sustains life, as previously stated, but also the process through which *our foods become endowed with life,* and, moreover, *the manner in which our tissues are built.*

The practical bearing of these discoveries is now made to appear: certain leucocytes (70 per cent of the aggregate of white cells) are scavengers merely because they convert food products, disease germs, broken-down cells, etc., into tissue cells. Now, when the vital process is below par, the body is vulnerable to disease: the scavenger cells are themselves unable to digest bacteria, and it is not the nutrient, tissue-forming granulations which they carry to all parts of the body, but living disease germs. In the light of Sajous' discoveries, when a patient is treated judiciously this can not happen, since, as previously stated, several of our well-tested remedies are able to raise the vital process to its fullest power; as the scavenger leucocytes form part of the body as a whole, they likewise acquire their full power when proper remedies are administered and are thus able to kill all bacteria they might ingest, convert them into tissue-granulations, and arrest disease.

Comparison with the prevailing doctrines shows a marked contrast. Pneumonia, for instance, is regarded by Osler as a "self-limited disease," which means that medicinal treatment is useless. In the light of Sajous' findings this is tantamount to a death certificate, since the pneumonia germs are thus allowed to multiply freely and kill the patient. He submits ample evidence attesting to the fact that pneumonia, as well as all other scourges of humanity, *can be checked by remedies which enhance the body's auto-protective functions.*

The germ-destroying leucocytes do not represent the only recourse available by the body when it is exposed to disease. These cells constitute only the first line of defense, as it were. The blood plasma itself, as shown by many investigators, is also a powerful bactericidal and antitoxic agent. But what is the origin of the substances which endow the fluid portion of the blood with these defensive properties? This constitutes another of Sajous' discoveries.

*Auto-antitoxin.*—As everyone knows, the mortality of diphtheria has been decreased to a remarkable extent since antitoxin has been used in its treatment. But the source of antitoxin in the body of the animal from whose blood it is obtained, as well as its chemical composition, has remained obscure. Sajous solved both of these problems. He showed that antitoxin contained (1) the oxygenized adrenal secretion (adrenoxidase) previously referred to, which, as the oxodizing (albuminous) constituent of the blood, is constantly present therein; (2) a ferment derived from the pancreas, trypsin; (3) a body rich in phosphorus, nucleo-proteid, derived from certain leucocytes; and (4) the thyroid secretion, which he termed thyroidase (opsonin). The adrenals, pancreas, leucocytes, and thyroid thus proved to be the source of diphtheria antitoxin—and, in fact, of all other antitoxins.

This suggested a line of research which brought out a discovery of even greater practical importance: If by inoculating an animal, the organs referred to could be caused to produce antitoxin by increasing the functional activity of these organs, could we not by means of our remedies also stimulate these organs, flood the blood with auto-antitoxin, and thus check a disease? A prolonged study of all the phases of the question enables Sajous to answer this question affirmatively and to formulate the general principle that "immunizing medication is the foundation of rational therapeutics"; in other words, that we should regard as the fundamental purpose of our efforts to cure disease, the use of remedies which, by increasing the functional activity of the organs that produce antitoxin, enhance correspondingly the bactericidal and antitoxic efficiency of the blood. This, he showed also, was the effect produced by those germs, toxins, poisons, etc., which are capable of evoking a defensive reaction in the body, as manifested by fever.

Sajous describes in detail all the diseases that are most fatal to mankind and shows conclusively that wherever cure had been effected by remedies, it was through agents which, by stimulating the organs referred to, increased the blood's asset in *auto-antitoxin* —the name given by him to the antitoxin which our body produces to antagonize disease.

But how does auto-antitoxin destroy pathogenic organisms, the toxins they secrete, poisons, toxic products of metabolism, etc.? Sajous shows that this differs in no way from the process of digestion, and that if auto-antitoxin is present in excess of the blood, the red corpuscles themselves may be digested (hemolysis). As to the process itself, the explanation he submits is based on the well-known fact that ferments are active, up to a certain limit, in proportion with the temperature to which they are exposed. When the temperature is normal, the ferments are active just sufficiently to carry on normal functions; when it is raised, their digestive activity is increased accordingly. Now, in the blood, the temperature is raised whenever its supply of adrenoxidase (the oxygenized adrenal secretion) and the nucleo-proteid granulations (supplied by leucocytes) is increased, owing to a reaction between the oxygen of the former and the phosphorus of the latter. Heat-energy being liberated in excess, the digestive activity of the ferments in the blood (which gives it its bacteriolytic and antitoxic properties) is correspondingly increased.

Yet, how are these germ-killing and poison-destroying substances caused to appear in the blood? How do poisons awaken the defensive reaction of the body?

*The Pituitary Body.*—Located on the very top of the spinal column in the sella turcica, immediately below the brain, protected on all sides with the utmost care, lies this organ. To its rôle in the economy a recently published text-book of physiology devotes seven lines! Indeed, beyond the fact that it is supposed to provide some sort of a secretion (the purpose of which has never been found), nothing is known as to the actual rôle of its anterior lobe; while its posterior lobe has been relegated to the rank of a vestigial remnant. Sajous demonstrates not only that this conception is false, but that the pituitary body in its relations to the functions of the body at large is even more important than the brain itself. None of these functions are impaired when the cerebral hemispheres of an animal are removed; all cease, however, when the whole pituitary body is submitted to the same ablation. The brain, as the organ of mind, can utilize the spinal system, with which it is connected, to execute its mandates; but the spinal system is also supplied with its own brain, the pituitary body, which Sajous terms the *"somatic brain,"* viz., the governing organ of all vegetative functions. He shows, moreover, that this "somatic brain" contains *a delicate organ whose mission is to protect the body against disease.*

In his first volume, Sajous had advanced the view that the anterior pituitary body was a sensitive organ which perceived, as it were, the presence of any adventitious substance in the blood. The existence of such a structure has since been confirmed independently in Europe, Gentès having found histologically therein a sensory structure resembling the olfactory area of the nasal cavity. Dr. Sajous' conception is readily explained: When the blood circulating in the anterior pituitary body contains any abnormal substance—a drug, a poison, a toxin, etc.—it affects this sensitive organ just as odoriferous particles do the olfactory area. Sajous studied this organ in the animal scale, through comparative morphology, and found it in all animals down to such low forms as mollusks, where it bears a suggestive name given to it by zoologists, viz., the *test-organ,* or *osphradium,* and is known by them to test the water ingested by these lowly beings.

But how, in the higher vertebrates, including man, does this test-organ protect against disease?

Sajous found, as previously stated, that the adrenals were provided with a center situated at the base of the brain. This, he subsequently ascertained, was a nucleus of cells in the posterior lobe of the pituitary body, which nucleus received nerve fibers from the sensitive test-organ referred to above. Now, the manner in which any poison or toxin can increase general oxygenation becomes apparent: it excites the test-organ, and this structure, in turn, through nerve-paths (passing by way of the base of the brain, the bulb, the cord, the sympathetic and splanchnic nerves) increases the functional activity of the adrenals. Thus the blood is provided with an excess of *adrenoxidase.* True, this is but one of the constituents of the body's protective substance, auto-antitoxin, but the manner in which the other components of the latter are formed is readily apprehended: the metabolism of all organs being rendered unusually active by the excess of adrenoxidase in the circulating blood, the formation of leucocytes is activated (leucocytosis) and the proportion of *nucleo-proteid,* their product, in the blood is correspondingly increased. The secretory activity of the pancreas being also stimulated by the excess of adrenoxidase, more *trypsin* is produced; and we thus have the three components of auto-antitoxin: the oxygen-laden adrenoxidase and the phosphorus-laden nucleo-proteid to supply the increased heat-energy required to enable the trypsin both in the phagocytes and in the blood to destroy bacteria, their toxins, or any other poisonous agent which the blood may contain.

This is not all, however; as shown by Sir A. E. Wright, the bacteria must be prepared for the phagocytic feast. Sajous, as previously stated, has discovered that the thyroid and parathyroids were the source of Wright's "opsonins," i. e., thyroidase. Now, the nucleus through which the adrenals receive their stimulating impulses from the test-organ is also shown by Sajous to transmit stimulating impulses to the thyroid apparatus. When disease germs invade the blood, therefore, their toxins, by exciting the test-organ, cause the blood to be provided with *thyroidase* (opsonin) to sensitize the germs, and with *auto-antitoxin* to destroy them.

The grave mortality from all diseases in the young as well as in the old shows, unfortunately, that although our body is endowed with protective functions, these are often inadequate to prevent, or even arrest, disease. This is where Sajous' labors are to prove most prolific in results, since they have demonstrated conclusively that *by means of the remedies in constant use among physicians,* the protective mechanism can be activated sufficiently to protect the patient. Pasteur's prophylactic treatment against rabies, Wright's inoculations, bacterial vaccines, etc., are but examples of the protection afforded through agents which stimulate the test-organ, this action differing in no way from that of the drugs referred to, the action of which can, besides, be more readily controlled. All these measures cause the blood to be flooded with thyroidase (opsonin) and auto-antitoxin. Hence the fundamental principle Sajous establishes—that *"immunizing medication is the foundation of rational therapeutics,"* which, as he shows by a comprehensive study of cancer, tuberculosis, syphilis, Asiatic cholera, cholera infantum, bubonic plague, epilepsy, puerperal eclampsia, and many other foes of mankind, is as applicable to the most virulent diseases as to the more benign. He not only points out the meaning of the *vis medicatrix naturæ,* but shows us how we can increase its efficiency and thus master disease.

Those who, like the writer, have availed themselves of Sajous' teachings in their daily work, have been able daily to appreciate the strength of his position, the power of the weapon or key he has placed in their hands, and the renewed confidence he thus inspires in practical medicine.

---

Applications for Furuncle and Mosquito Bites.—Gallois prescribes a solution of metallic iodine in acetone (40 per cent) to abort boils and to relieve the irritation following bites of insects, especially mosquitoes. It forms a sort of varnish in drying, which relieves the itching and checks inflammation. Generally one application is sufficient, but it may be repeated at the end of five or six hours. The discoloration lasts about twenty-four hours, and is easily removed with soap and water. A paste of cigar ashes mixed with a drop of water is often effective, or the bite may be rubbed with a piece of dry soap. In the last two methods, it is the alkaline salts which relieve the itching.—*Bulletin général de thérapeutique.*

---

An easy way to straighten out a probe that has been much bent and twisted, is to roll it under the foot on an even floor.—*American Journal of Surgery.*

---

Fluids in Chronic Interstitial Nephritis.—Colliver does not believe in "flushing the kidneys" in nephritis, and says that much benefit is often derived by restricting the amount of liquid ingested. He sums up his position by saying: Before compensation is broken, ingestion of fluids has marked effect and tends to hasten the crisis. After compensation is broken, ingestion of fluids is of no advantage to the kidneys, but tends to embarrass the heart and to increase effusion and edema. The amount of fluids ingested should be slightly in excess of the amount of urine excreted.—*California Medical and Surgical Reporter.*

